# Inhibition of DNA-repair genes Ercc1 and Mgmt enhances temozolomide efficacy in gliomas treatment: a pre-clinical study

**DOI:** 10.18632/oncotarget.4909

**Published:** 2015-08-20

**Authors:** Sandra G. Boccard, Sandie V. Marand, Sandra Geraci, Laurie Pycroft, François R. Berger, Laurent A. Pelletier

**Affiliations:** ^1^ Univ. Grenoble Alpes, Grenoble Institut des Neurosciences, GIN, F-38000 Grenoble, France; ^2^ CHU de Grenoble, F-38000 Grenoble, France; ^3^ Oxford Functional Neurosurgery and Experimental Neurology, University of Oxford, UK

**Keywords:** DNA-repair, chemoresistance, glioma, Temozolomide (TMZ), siRNA

## Abstract

Gliomas are the most common primary brain tumors. To date, therapies do not allow curing patients, and glioblastomas (GBMs) are associated with remarkably poor prognosis. This situation is at least partly due to intrinsic or acquired resistance to treatment, especially to chemotherapy. In 2005, temozolomide (TMZ) has become the first chemotherapeutic drug validated for GBM. Nevertheless TMZ efficacy depends on Mgmt status. While the methylation of Mgmt promoter was considered so far as a prognostic marker, its targeting is becoming an effective therapeutic opportunity. Thus, arrival of both TMZ and Mgmt illustrated that considerable progress can still be realized by optimizing adjuvant chemotherapy. A part of this progress could be accomplished in the future by overcoming residual resistance. The aim of the present study was to investigate the involvement of a set of other DNA-repair genes in glioma resistance to temozolomide. We focused on DNA-repair genes located in the commonly deleted chromosomal region in oligodendroglioma (1p/19q) highly correlated with patient response to chemotherapy. We measured effects of inhibition of ten DNA-repair genes expression using siRNAs on astrocytoma cell response to cisplatin (CDDP) and TMZ. SiRNAs targeting *ercc1*, *ercc2*, *mutyh*, and *pnkp* significantly sensitized cells to chemotherapy, increasing cell death by up to 25%. *In vivo* we observed a decrease of subcutaneous glioma tumor growth after injection of siRNA in conjunction with absorption of TMZ. We demonstrated in this pre-clinical study that targeting of DNA-repair genes such as Ercc1 could be used as an adjuvant chemosensitization treatment, similarly to Mgmt inhibition.

## INTRODUCTION

Malignant gliomas are the most common type of primary tumors of the central nervous system in Europe and US, accounting for 80% of patients [1, 2]. Annual incidence of these neoplasms is approximately 4–7 *per* 100,000 in men and 3–5 in women. The number of patients is expected to increase in industrialized countries as the population ages. Tumor diagnoses are based on histopathological features and are graded according to the World Health Organization classification [3]. Among them, glioblastomas multiform (GBM) are the most frequent, and harbor the poorest prognosis [1, 2] as only 2 to 5% of patients survive after 2 years [4]. These gliomas are indeed highly refractory to any treatment.

Since the publication of the phase III study initiated by the European Organisation for Research and Treatment of Cancer (EORTC) and the National Cancer Institute of Canada Clinical Trials Group (NCIC CTG) groups in 2005, standard treatment is surgery followed by radiotherapy and temozolomide (TMZ)-based chemotherapy [5]. Concomitant radiotherapy and TMZ administration has significantly improved median survival of glioma patients, from 12.1 to 14.6 months, with 27% of patients alive at 2 years, instead of 10% without TMZ [6]. This improvement was confirmed during the five-year follow-up period with a median survival of 9.8% *versus* 1.9% [7]. The benefit offered by TMZ has been established in a systematic review [8]. Nevertheless, malignant gliomas prognosis remains poor and most of the patients relapse, thus there is a clear need for new treatments.

Some glioma hallmarks were demonstrated to be of clinical relevance and may offer opportunities for new therapies. Among them, some are responsible for a poorer prognosis (mutation/amplification of the epidermal growth factor receptor (EGFR) [9, 10]). and, on the other hand, some others are associated with a better one (O-6-methylguanine-DNA methyltransferase (MGMT) promoter methylation [11], mutations in isocitrate dehydrogenase (IDH) genes [12] and 1p/19q deletion [13]). The loss of heterozygosity (LOH) of these chromosomal arms was indeed correlated with the oligodendroglioma noticeable chemosensitivity [13, 14], suggesting that some key genes involved in chemosensitivity may be located on this area.

This fact consequently provides a list of more than 1,700 putative target genes to increase glioma sensitivity to chemotherapy, and among them some of the 150 DNA-repair genes inventoried by Wood et al. [15].

Prior to 2005, GBM patients had been treated for decades with chemotherapy agents. The failure of these drugs showed that intrinsic or acquired resistance mechanisms are of paramount importance in these high grade tumors. Since most drugs were alkylating agents, inducing a panel of DNA damages, DNA repair is a highly relevant resistance mechanism. Moreover, the DNA repair gene *Mgmt* was shown to be associated to patients' outcome when treated with TMZ [11]. Mgmt was considered as a prognostic marker as well as a therapeutic opportunity [16]. Unfortunately, targeting Mgmt activity using chemical inhibitor O6BG was first associated with a crippling hematopoietic toxicity [17, 18]. Yet innovative strategies have emerged, allowing for reduction of side effects and transforming the hypothesis into medical practice [19].

We proposed the hypothesis that other DNA-repair genes would be involved in glioma resistance to TMZ. Using small interfering RNA (siRNA) we found that inhibition of 4 of them, namely *ercc1*, *ercc2*, *mutyh* and *pnkp*, showed a significant impact on cisplatin (CDDP) and/or TMZ cytotoxic effect in several human glioma cell lines. Furthermore, an ercc-1 siRNA-based adjuvant treatment was able to improve the efficacy of TMZ in glioma tumor bearing mice. This study offers a promising adjuvant therapy to improve the clinical management of malignant gliomas.

## RESULTS

### *In silico* identification of candidate genes

To select genes potentially involved in glioblastoma chemoresistance, the chromosomal areas commonly considered as correlated with oligodendroglioma chemo-sensitivity (1p36–1p32 and 19q13.2–19q13.4; [20]) were screened, using databases on the web (http://www.ncbi.nlm.nih.gov/PubMed/; http://www.ensembl.org; http://www.cgal.icnet.uk/DNA_Repair_Genes.html; Supplementary to the paper by Wood, 2005) and published data [15, 21, 22]. Nine genes located in these regions belong to different DNA-repair systems among the 1,700 genes (Table 1).

**Table 1:** List of DNA repair-associated genes located on 1p/19q LOH regions

DNA repair genes	DNA repair system	Reference	Location
ercc1	NER	NM_001983	19q13.2–3
lig1	NER	NM_000234	19q13.2–3
ercc2	NER	NM_000400	19q13.3
pold1	NER and MMR	NM_002691	19q13.3
ruvbl2	HR	NM_006666	19q13.3
pnkp	BER	NM_007254	19q13.3–4
rad54L	HR	NM_003579	1p32
mutyh	BER	NM_012222	1p34.3
mad2L2	ADN polymerase	NM_006341	1p36

According to Wood *et al,* 2005

NER, nucleotide excision repair; MMR, mismatch repair; HR, homologous recombination; BER, base excision repair.

### Screening of DNA-repair genes involved in chemoresistance of astrocytoma cells

**Figure 1:**
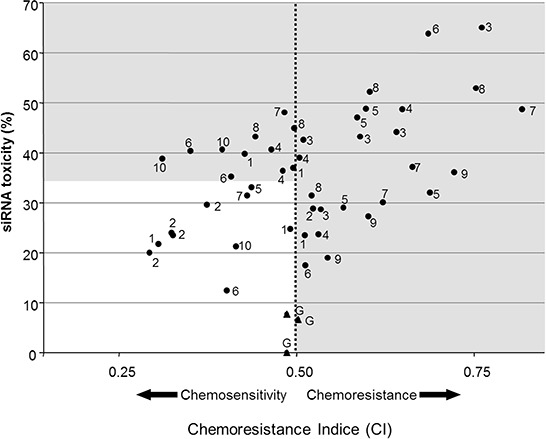
Efficiency and toxicity of siRNAs Cell viability (%) was measured in the presence (x-axis) and absence (y-axis) of CDDP (5.10^−6^ M). SiRNA (150 nM) inducing less than 35% of cell death without drug, and a chemosensitization were selected for further analyses.

The *in vitro* part of the project was developed as a 3-stage strategy. SiRNAs targeting candidate genes were screened on one cell line (U373). We chose to achieve this step using CDDP because Ercc1 is associated with a chemotherapy resistance (especially to platinum drugs) in several experimental models (see discussion). Then, the study was extended to six astrocytoma-derived cell lines and validated at the molecular level. Finally, the study was expanded to TMZ. Nine DNA-repair genes were screened on the U373 cell line with up to 5 different siRNAs *per* gene (Table 1). Cell viability was measured in the absence and presence of CDDP. SiRNAs were selected on the basis of two criteria: the absence of basal toxicity and their ability to improve chemotherapy treatment. SiRNAs inducing more than 35% cell death in absence of drug were considered as toxic and discarded. On the other hand, those increasing sensitivity to the chemotherapy drug were retained. An siRNA was considered as chemosensitizing when its chemoresistance index (CI) was lower than the GFP siRNA's CI (0.5). In Figure 1, the white square corresponds to siRNAs with low toxicity and chemosensitization properties. Nine siRNAs out of 46 matched with these criteria, corresponding to 6 genes: ercc1, ercc2, mutyh, pnkp, ruvbl2 and pold1.

Highlighted genes were further analyzed to validate preliminary screening, using only the most efficient siRNA for each gene. The experiments were extended to 5 other cell lines and we confirmed that 4 siRNAs, ercc1#2, ercc2#1, mutyh#5 and pnkp#1, had significant chemosensitization effects (Table 2). Inhibition of ercc1 was the most effective in sensitizing cells to CDDP (up to 24.9%). Moreover, its effect was the most widespread since 4 out of the 6 cell lines were sensitized. The knock-down induced by the 3 other siRNAs was equally effective (up to 17%) but only on 2 (mutyh and pnkp) or 3 (ercc2) cell lines.

**Table 2:** SiRNA-induced drug sensitization *in vitro*

Drug	Cell line	Ercc1	Ercc2	Mutyh	Pnkp
CDDP	U373	19.9**	15.8**	16.9**	17.6 **
	GHD	24.9**	10.1*	10.8*	12.1*
	LN229	24.7**	16.9**	*n.s.*	*n.s.*
	U318	14.1**	*n.s*	*n.s.*	*n.s.*
TMZ	U373	24.5**	17.6**	26.0**	25.1**
	GHD	9.5**	*n.s*	*n.s.*	*n.s.*

Drug sensitization index (DS) was measured for the 4 siRNA candidates on 6 cell lines (U373, U87, GHD, LN229, U138 and CCF). Data are the mean of 3 independent experiments. Only significant values are indicated. n.s., non-significant.

In order to validate these effects at molecular level, siRNA-induced down-regulation of the mRNAs was measured (Table 3). The target specificity was confirmed by the absence of an effect on either cyclophiline A or other gene expression (*data not shown*). There was no obvious link between the efficiency of siRNA in decreasing mRNA levels (nor with residual mRNA content; data not shown) and functional impact on cell viability. This is reinforced by the observation that the *ercc1* mRNA level actually decreased (by 65%) in U87 cells which were not yet chemosensitized.

**Table 3:** siRNA-induced downregulation of mRNA measured by RT-qPCR

Cell line	Quantified mRNA	Targeted siRNA		siRNA GFP		Inhibition (%)	*p*-value (ANOVA)
		mean (fMol)	sem	mean (fMol)	sem		
GHD	ercc1	8.6E-06	2.0E-06	2.1E-05	5.0E-06	58.6	0.019
	ercc2	1.7E-07	3.9E-08	4.3E-07	7.1E-08	61.6	0.011
	mutyh	5.0E-05	1.3E-05	1.7E-04	3.6E-05	71.0	0.004
	pnkp	8.0E-06	1.6E-06	5.0E-05	1.6E-05	83.8	0.005
U373	ercc1	1.9E-06	8.2E-07	8.4E-06	2.3E-06	77.4	0.018
	ercc2	1.2E-07	3.0E-08	2.8E-06	7.9E-07	95.7	0.003
	mutyh	2.6E-05	7.6E-06	9.5E-05	1.8E-05	72.9	0.007
	pnkp	3.6E-05	9.6E-06	6.5E-05	1.6E-05	44.2	0.013
	mgmt	4.2E-06	5.2E-07	2.1E-05	1.0E-06	80.4	0.001

All used siRNAs significantly reduced expression of their respective targets, when compared to the control siRNA (GFP). Measures were performed twice from each cell sample and results were expressed as the mean of at least three independent samples (originating from independent *in vitro* experiments)

### Sensitization to temozolomide

**Figure 2:**
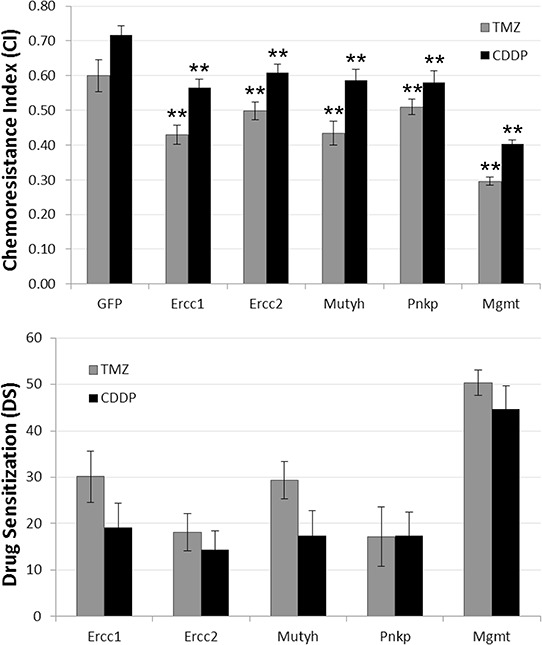
SiRNA-induced chemosensitization U373 cells were transfected with siRNA targeting GFP, Ercc-1, Ercc-2, Mutyh or Pnkp, and treated with CDDP (grey) or TMZ (black). **A.** The Chemoresistance Index (CI) corresponds to the proportion of a cell population that survived chemotherapy. It was computed as follows: cell number with chemotherapy / cell number (OD) in control condition. **B.** DS corresponds to the chemosensitivity induced by siRNA. Data represented the mean of 3 independent experiments. Statistically significant difference between effects induced by siRNA GFP and other siRNA were indicated by 2 asterisks (*p* < 0.01). Error bars represent the bootstrapped standard errors.

Since TMZ is the gold standard in chemotherapy for gliomas, the study was extended to this drug using GHD and U373 cell lines. All siRNAs sensitized U373 cells to both CDDP and TMZ with a similar efficacy (Figure 2). The siRNAs were less efficient on GHD cells treated with TMZ compared to CDDP and only the effects of the ercc1 siRNA remained statistically significant (*data not shown*).

### *In vivo* therapeutic effect of Ercc1-siRNA

**Figure 3:**
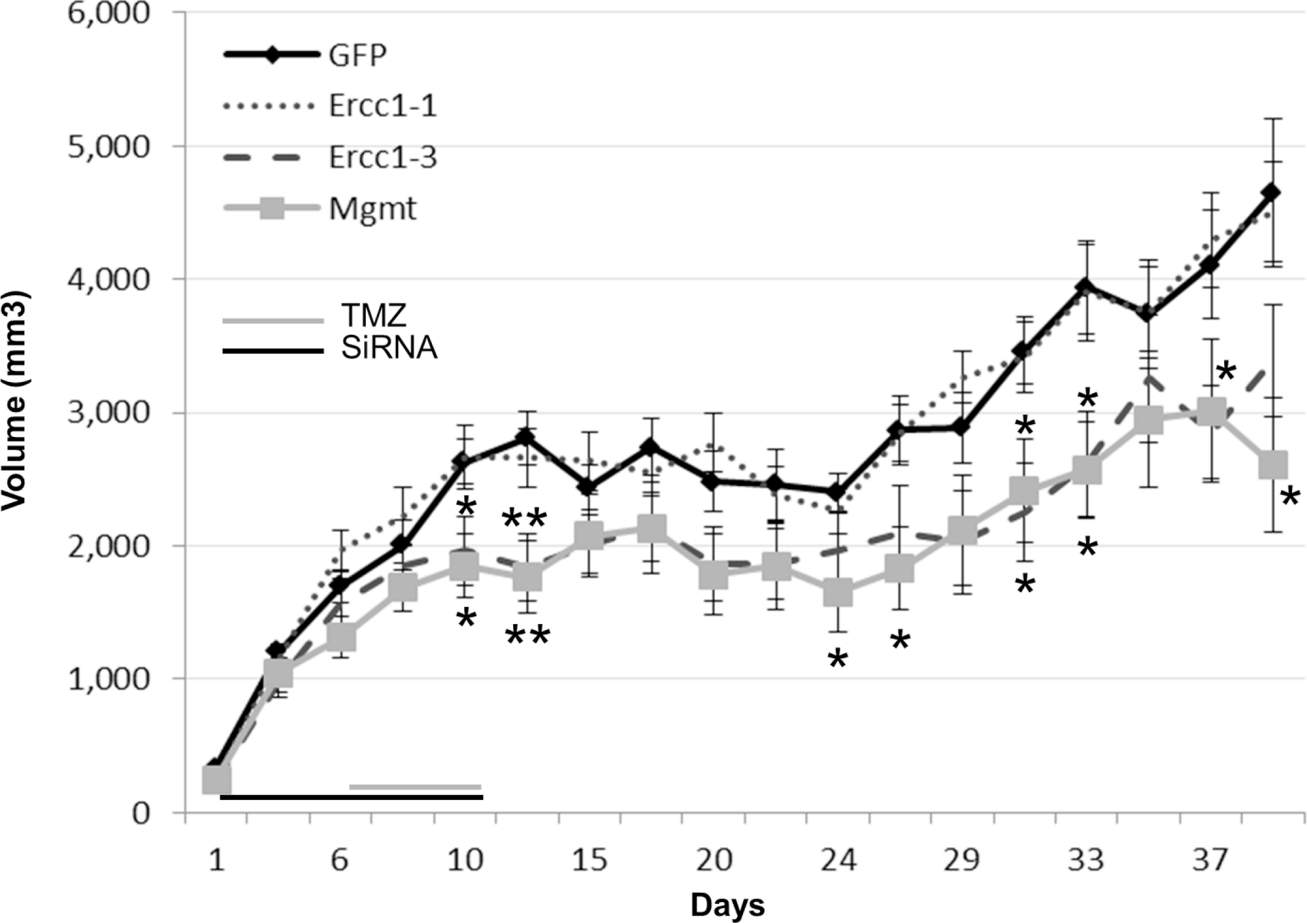
Therapeutic effects of the combined effect with TMZ and Ercc1-siRNA *in vivo* *In vivo* combined effects of Ercc1#1-, Ercc1#3- or Mgmt-siRNA (4 μg) with TMZ (4.2 mg/kg) on U373 xenografts growth. The combined therapy with TMZ and Ercc1#3 (dash line) provided a significantly stronger reduction of tumor growth than TMZ with a control siRNA (GFP) (*, *p* < 0.05). The first day of administration was defined as ‘day 0′. The days on which the treatments were administered are indicated by grey (TMZ) and black (siRNA) lines at the bottom of the graphs. Mean tumor volumes +/− SE are shown. 2-tailed Student's *T*-tests were used to evaluate results significance.

The chemosensitive effect of siRNA targeting Ercc1 was assessed in *Nude* mice carrying human glioma xenografts in 3 independent experiments. In all of them, Ercc1-siRNA #3 was highly effective in inhibiting tumor growth. Its effect was significantly different from the control treatment (GFP-siRNA) and greater than a siRNA targeting Mgmt (Figure 3). None of the groups experienced side effects such as body weight loss.

## DISCUSSION

Despite the demonstration of the improvement in survival following radio- and chemotherapy regimens (TMZ) for glioblastoma, this malignancy remains an incurable disease.

A variety of cancer cell resistance mechanisms have been described, including decreased drug uptake, increased drug efflux, intracellular drug inactivation or drug-induced damage repair. The high activity of these mechanisms in gliomas have hampered the use of chemotherapy and thereby prevented widespread its acceptance as an effective treatment modality. Most chemotherapy drugs commonly employed in the past such as the PCV regimen (Procarbazine, Lomustine (CCNU), and Vincristine), CDDP, fotemustine or the presently used TMZ are DNA-alkylating molecules which damage DNA. These data have led many studies to focus on the link between DNA-repair and chemoresistance [23–26]. However it is only recently that the response to TMZ was found strongly correlated with *mgmt* promoter methylation status [11]. Down-regulation of Mgmt expression would explain sensitivity to therapy due to the absence of alkylating adducts repair. Mgmt is considered as a major DNA-repair enzyme involved in resistance to several chemotherapy drugs since the end of the twentieth century [27]. Hegi *et al*. have shown a correlation between the DNA-repair gene *mgmt* and response to TMZ [11]. Indeed, several clinical trials have addressed the possibility of increasing chemotherapy efficacy while blocking mgmt activity [28–31]. Using an innovative strategy, Adair *et al.* have recently demonstrated that Mgmt blockade is associated with an improvement of TMZ efficacy [19]. Although these results are preliminary with a small cohort of patients, they consist of the first dual clinical and functional demonstration of the link between Mgmt and TMZ resistance. These results indicate that DNA-repair inhibition is a highly attractive and plausible therapeutic avenue.

Thus Mgmt is to date the main molecular pathway related to TMZ response in glioblastoma.

As discussed above, the decrease in *mgmt* expression has often been reported, as a consequence of its promoter methylation. In this study, we observed both that siRNAs were responsible for a decrease of expression and a chemosensitizing impact. Similar observations were made using a second siRNA duplex (data not shown). Our results suggest that mgmt expression was sufficient for supporting a significant DNA repair activity.

As discussed below, we demonstrated that targeting other DNA-repair genes can provide similar efficacy compared to Mgmt invalidation.

### Gliomas cytogenetic and clinical management

In contrast to glioblastomas, oligodendrogliomas constitute a chemoresponsive entity. The correlation between 1p/19q LOH and oligodendroglioma chemosensitivity [13] prompted us to perform an *in silico* analysis of these chromosomal areas in order to identify genes potentially involved in chemosensitivity. Jenkins *et al*. demonstrated that 1p/19q LOH was mediated by a translocation [32]. This early cytogenetic event was also associated with a longer overall survival. These data led to a better understanding of the cytogenetic alterations mechanisms frequently associated. But, above all, they reinforce the hypothesis that expression of genes located in both regions can be altered, being finally responsible for chemosensitivity of oligodendroglioma.

Among the 1,700 genes located on the 1p and 19q chromosomal areas commonly considered as correlated with oligodendroglioma chemosensitivity, we found 9 DNA-repair genes. We confirmed the *in silico* analysis with a functional genomic approach using siRNAs to knock down gene expression, mimicking LOH.

### *In vitro* study

For validating candidates at functional level, we developed an adequate *in vitro* chemosensitivity assay in 96-well plates to test all the designed siRNA. The temporal sequence of treatments was crucial since the siRNA impact on DNA repair activity had to line up with the chemotherapy treatment. We succeeded in interfering with drug-induced DNA adduct repair using an siRNA transfection performed 24 hours prior to chemotherapy. This result is consistent with data previously published by Parker *et al*. showing that drug effect occurs in the first 6 hours of treatment [33].

SiRNAs found to induce cellular toxicity were discarded from the study to prevent erroneous conclusions. The observed toxicity could be the result of a critical gene inhibition and, though possibly interesting, it would have masked any intended chemosensitization. Similarly, siRNAs that did not have any impact on cell numeration were rejected. In the end, only 9 out of the 46 tested sequences in this study had a significant chemosensitivity impact, demonstrating the importance of a validated assay to bring out efficient siRNA targets.

The most prominent functional impact was observed with the two most studied genes of the NER system: *ercc1* and *ercc2*, a.k.a. xeroderma pigmentosum group D (XPD). The NER system has a critical role in repairing DNA and has been extensively studied in cancer cells.

The most important chemosensitization occurred when *ercc1* expression was inhibited: Sensitization was not larger but was found in a greater number of cell lines (4 *vs* 3 for *ercc2*, and 2 for *mutyh* and *pnkp*). These results emphasize previous reports on the major role of ercc1 in repairing DNA alterations related to alkylating chemotherapy. Thanks to its nuclease activity, the ercc1 protein plays a crucial role in the early excision step of damaged DNA [34, 35]. It is involved in CDDP-induced adduct repair *in vitro* [36, 37]. Reduction of its expression enhances CDDP cytotoxicity in ovarian cancer cell lines [38, 39], and was generally found involved in drug resistance of cultured cells [40, 41].

Moreover, its expression or polymorphisms were correlated with stage or clinical outcome in several cancers e.g. glioma [42], stomach [43], ovary [44] and lung [45, 46], (for review, see: [47, 48]). Thus, it appears as a very consistent if not universal component of tumor drug resistance and thus as a spread diagnostic/prognostic marker and/or therapeutic target.

*Ercc2* expression was correlated with resistance to alkylating compounds in numerous cell lines [49, 50], including glioma cell lines [51], but no link has been established between this expression and NER activity [50]. This suggests that ercc2 is not an NER rate-limiting enzyme as in primary lymphocytes [52]. It could also act *via* the homologous recombination repair system as in SKMG-4 glioma cells [53]. Another level of complexity is the number of *ercc2* polymorphisms without clear associated phenotypes [54]. These polymorphisms obviously contribute to governing ercc2 cellular function. So, while a previous report showed that its overexpression increased DNA repair in a glioma cell line [55], the present results are the first demonstration of its functional involvement in drug resistance.

We also found two genes belonging to the BER system: *mutyh* and *pnkp*. Only the systematic bio-informatics analysis prompted us to analyze them, as no previous publication involved them in chemosensitivity.

Mutyh is known to repair 8-oxo-7,8-dihydro2′deoxyguanosine (8-oxodG) caused by oxidation. Gene mutations and variants were associated with development of multiple colorectal adenomas and cancers [56]. This is the first time that mutyh has been implicated in chemosensitivity, warranting further studies to investigate its mechanisms of action.

Pnkp was shown to be involved in repairing DNA strand breaks caused by reactive oxygen species, ionizing radiation or alkylating agents [57, 58]. It has been related to susceptibility to genotoxic agents but not to chemosensitivity [59]. Nevertheless, interactions of pnkp with another DNA-repair protein such as Xrcc1, which is related to tumoral processes, could account for our results [60].

### Heterogeneity

We observed heterogeneity in responses to siRNAs among the 6 cell lines. While no siRNA improved chemotherapeutic effects on CCF and U87, all siRNAs sensitized U373 to both CDDP and TMZ. Such heterogeneous results were already observed for glioma cell lines in an unrelated study [61]. These differences were not related to known differences in cytogenetic or genetic status e.g. p53 mutations. Heterogeneity in cell line response to siRNAs may result from the strong variability in gene expression. However, we did not find any correlation between the mRNA levels (in basal or CDDP conditions) and the siRNAs' ability to chemosensitize. Such a correlation was not either found for Mgmt. Indeed, while the methylation status of the promoter is a routinely used marker of response to TMZ, *Mgmt* expression failed to be as useful [62]. It is important to note that among cell lines with opposite behaviors, U373 and U87 are the most frequently used for studying gliomas, as shown by the number of publications (search «glioma»» and «U87» or «U373» or «U138» or «LN229» or «CCF»: 1,534; 513; 63; 127; 94 respectively on Pubmed in March 2015). Thus, no cell line can be sufficiently representative for constituting an *in vitro* model, and results obtained with only one cell line should be considered very cautiously for clinical applications.

### SiRNA

The delivery of drugs to the central nervous system (CNS) is a crucial issue in medical management. We and others have demonstrated the poor capacity of siRNAs packaged with polyethylenimine (PEI) to pass through the blood brain barrier [63–65] and among the numerous other vectors or strategies investigated for targeting brain and intracranial tumors, none has been validated to date. This is the reason why we chose to use ectopically implanted tumor instead of intracranial ones. As a second consequence of using siRNAs in this study, our results cannot be easily transferred to bedside in absence of a validated procedure at the clinical level. Novel chemical agents may be developed that will enable translation of this technique to clinical practice. Indeed, the Ercc1-XPF interaction can be successfully prevented *in vitro* [66, 67], providing hope for future clinical use. But the development of chemical inhibitors directed to Ercc2, Mutyh and Pnkp proteins is needed.

The aim of our study was to elucidate a part of the mechanisms of astrocytoma drug resistance. We have identified and functionally validated that Ercc1, Ercc2, Mutyh and Pnkp participate to TMZ resistance in gliomas. Our results suggest that survival of glioma patients may be improved when targeting these genes.

## MATERIALS AND METHODS

### Cell lines and medium

U373, U138, U87, CCF and LN229 cell lines derived from primary human astrocytomas, purchased from American Type Culture Collection (ATCC, Rockville, MD), maintained in DMEM (Cambrex Biosciences, New Jersey, USA) supplemented with 10% fetal calf serum (v/v; AbCys, Paris, France). Cells were maintained in 5% CO_2_ at 37°C in a humidified incubator.

Since the experiments were performed the ATCC found that the U251 and U373 cell lines had a common origin [68]. We then cannot be sure of the cell-line we actually worked on. Nevertheless, both cell-lines derived from glioblastomas.

The GHD cell line was obtained in our laboratory from a human glioblastoma (genotype was checked with fluorescence *in situ* hybridization and contained chromosome 7 polysomy and chromosome 10 monosomy).

### Inhibition of gene expression by siRNA

SiRNAs (three to five *per* gene) were designed (Table 4) and delivered in duplex form (Eurogentec, Belgium). SiRNA targeting Green Fluorescent Protein (GFP) were used as a control. *In vivo*-jetPEI® was from Polyplus-transfection (Illkirch, France)

**Table 4:** Sequences of siRNAs

Gene	SiRNA #1	SiRNA #2	SiRNA #3	SiRNA #4	SiRNA #5
ERCC1	5′-AUC-CCG-UAC-UGA-AGU-UCG-U-3′	5′-GGA-GCU-GGC-UAA-GAU-GUG-U-3′	5′-CAA-GGC-CUA-UGA-GCA-GAA-A-3′	5′-ACA-GCU-CAU-CGC-CGC-AUC-A-3′	5′-AGA-GAA-GAU-CUG-GCC-UUA-U-3′
ERCC2	5′-GGA-CGU-CGA-UGG-GAA-AUG-C-3′	5′-AGA-CGG-UGC-UCA-GGA-UCA-A-3′	5′-UCA-UCA-UCG-AGC-CCU-UUG-A-3′	5′-GGA-ACA-AGC-UGC-UCU-UUA-U-3′	5′-UGA-CUU-UCU-UAC-CUU-CGA-U-3′
GFP	5′-GAC-GUA-AAC-GGC-CAC-AAG-UUC-3′		/	/	/
LIG1	5′-AGA-CGC-UCA-GCA-GCU-UCU-U-3′	5′-GAA-GAU-AGA-CAU-CAU-CAA-A-3′	5′-AGA-CAG-CAG-AGG-CCA-GAA-A-3′	5′-GCA-GAC-GUU-CUG-CGA-GGU-U-3′	5′-GCA-GAU-CCA-GCC-AUU-CCA-A-3′
MAD2L2	5′-GAA-GAA-UGA-UGU-GGA-GAA-A-3′	5′-GAC-UCG-CUG-UUG-UCU-CAU-G-3′	5′-CUC-GCA-ACA-UGG-AGA-AGA-U-3′	5′-GAA-GAU-CCA-GGU-CAU-CAA-G-3′	5′-UGA-GCA-GGA-UGU-CCA-CAU-G-3′
MGC13170	5′-CAA-GGA-CUU-GGC-UGC-UGA-G-3′	5′-GGA-GAA-GGU-GGA-UAA-GUG-G-3′	5′-GAA-GGU-GGA-UAA-GUG-GGC-U-3′	/	/
MUTYH	5′-GAA-GCA-UGC-UAA-GAA-CAA-C-3′	5′-UGG-GAU-GAU-UGC-UGA-GUG-U-3′	5′-GCA-CCC-UUG-UUU-CCC-AGC-A-3′	5′-GGU-UGU-CCA-CAC-CUU-CUC-U-3′	5′-GCU-GAC-AUA-UCA-AGU-AUA-U-3′
PNKP	5′-CAC-ACU-GUA-UUU-GGU-CAA-U-3′	5′-AGA-GAC-CCG-CAC-ACC-AGA-A-3′	5′-GAA-UCU-UGU-ACC-CAG-AGA-U-3′	5′-AGU-CCA-CCU-UUC-UCA-AGA-A-3′	5′-CAA-CCG-GUU-UCG-AGA-GAU-G-3′
POLD1	5′-GGA-GAU-GGA-GGC-AGA-ACA-C-3′	5′-GUU-GGA-GAU-UGA-CCA-UUA-U-3′	5′-UCA-CCG-GUU-ACA-ACA-UCC-A-3′	5′-CUU-AGA-CUC-CAC-CAG-CUG-C-3′	5′-AUU-CAG-AUG-GGA-UAC-CUC-C-3′
RAD54L	5′-CCA-GCA-UUG-UGA-AUA-GAU-G-3′	5′-UCA-CCU-CGC-UAA-AGA-AGC-U-3′	5′-GGA-GCU-GUU-UAU-CCU-GGA-U-3′	5′-UGA-UCU-GCU-UGA-GUA-UUU-C-3′	5′-GCA-GUG-AGA-CCC-AGA-UCC-A-3′
RUVBL2	5′-AUC-UUC-UCC-CUG-GAG-AUG-A-3′	5′-ACU-GAC-CCU-CAA-GAC-CAC-A-3′	5′-ACG-CAA-GGG-UAC-AGA-AGU-G-3′	/	/

For *in vitro* experiments, cells were transfected with siRNA duplexes at a concentration of 150 nM in the culture medium by using Oligofectamine (Invitrogen, Cergy-Pontoise, France) 24 h after cell seeding according to the manufacturer's instructions. Each condition (siRNA) was tested in 3 independent experiments, with six replicates each time.

For *in vivo* delivery, siRNA were diluted in 5% glucose (final) using 4 μl of glucose stock solution per μg of siRNA. 0.1 μl of *in vivo*-jetPEI® were used *per* μg of siRNA, and were diluted in the same volume of glucose stock solution. The transfection reagent solution was added to the siRNA solution and incubated for 15 min at room temperature. The mixture was diluted in 200 μl (final) of water.

### Evaluation of siRNA effect on drug sensitivity

We developed an *in vitro* assay designed to screen siRNAs. Cells were seeded in 96-well plates. SiRNAs (150nM) were transfected the second day. The next day, cells were treated with drugs: CDDP (5.10^−6^ M) (Merck, NJ, USA) or TMZ (10 mg/ml) (Schering-Plough, Levallois-Perret, France) and post-incubated with a drug-free medium for 96 hours. Cell survival was then evaluated by measuring mitochondrial succinate dehydrogenase activity with 3-(4,5-Dimethylthiazol-2-yl)-2,5-diphenyltetrazolium bromide (MTT; Sigma; 0.5 mg/ml) added to the culture medium. Culture medium was discarded after 4 h and formazan crystals were dissolved in DMSO/ethanol (50/50). Optical density (OD) was read at 540 nm.

Chemoresistance was related to an index (CI) corresponding to the proportion of a cell population that survived chemotherapy. It was computed as follows: cell number (OD) with chemotherapy / cell number (OD) in control condition. The benefit of transfection was represented by the siRNA-induced drug sensitization index (DS), which corresponds to the cell population (%) that survived a simple chemotherapy treatment but died in response to the same treatment following siRNA transfection. It was computed as follows: (CI _siRNA GFP_ – CI _siRNA X_) /CI _siRNA GFP_ × 100. Standard errors of this index were computed using a bootstrap, technique implemented in the boot library [69] in R [70]. Significant differences between series were tested by ANOVA using Statview (SAS institute). Differences were considered significant when *p* < 0.05 (*) and highly significant when *p* < 0.01 (**).

### Real-time quantitative reverse transcription-PCR

The NucleoSpin kit (Macherey-Nagel, Germany) was used to extract RNA and cDNA was purified after reverse transcription (Mini Elute, PCR Purification Kit, Qiagen, France).

Real-time quantitative PCR was performed on a Light Cycler (Roche Diagnostic, France) using SYBR Green.

Measures were performed twice from each cell sample and results were expressed as the mean of at least three independent samples (originating from independent *in vitro* experiments). Specific primers (Eurogentec, Belgium) were as follows: Cyclophiline A: forward primer, 5′-TTC ATC TGC ACT GCC AAG AC-3′; reverse primer, 5′-TCG AGT TGT CCA CAG TCA GC-3′; Ercc1: forward primer 5′-GGC GAC GTA ATT CCC GAC TA-3′; reverse primer, 5′-AGT TCT TCC CCA GGC TCT GC-3′; Ercc2: forward primer, 5′-CGG AAC TAT GGG AAC CTC CT-3′; reverse primer, 5′-TAC TTC TCC AGG GCG ACA CT-3′; Mutyh: forward primer, 5′-GTC CTG ACG TGG AGG AGT GT-3′; reverse primer, 5′-CCT CTG CAC CAG CAG AAT TT-3′; Pnkp: forward primer, 5′-TCG AGA GAT GAC GGA CTC CT-3′; reverse primer, 5′-TTT ATT GTG GAG GGG AGC TG-3′; Mgmt: forward primer, 5′- AGCTGATGCCGTGGAGGT-3′; reverse primer, 5′- ACGACTCTTGCTGGAAAACG-3′.

### Ethics and animal care

All procedures related to animal care are conform to the guidelines of the French government and were approved by our institutional ethics committee (authorization n° 201503261330924). All animals were Nude mice (Harlan, France) of 5 weeks-old upon arrival.

### Human xenografts assays

The U373 glioma cells (ATCC, MD, USA) were subcutaneously implanted one week after the mice arrival. A 100 μL solution containing 0.75 × 10^6^ U373 cells in DMEM were injected subcutaneously (s.c.) into the upper leg of nude mice (Charles River). When tumors are visible, mice were randomized into groups of 6 mice each. Ercc1, Mgmt or GFP siRNA were injected daily into the peritoneal cavity of each mouse on Days 20–29 (4 μg siRNA in 5% final Glucose 200 μL of H20). TMZ treatment began on the 5^th^ siRNA administration day. It was orally administrated daily for 5 days at a dose of 4.2 mg/kg in 200 μL of orange juice. Tumors were measured every other day with a dial caliper. Results are presented as the mean ± SE with significance calculated by 2-tailed Student's t test. Significance was assigned for a *p*-value < 0.05.
